# Supplementary material for: Investigating hospital Mycobacterium chelonae infection using whole genome sequencing and hybrid assembly
Source: PLoS One. 2020 Nov 9;15(11):e0236533. doi: 10.1371/journal.pone.0236533 (PMC7652343; doi:10.1371/journal.pone.0236533)
Supplement: Attachment — Submitted filename: PONE-D-20-20588_reviewer.pdf [file pone.0236533.s005.pdf]

PLOS ONE

Investigating hospital <i>Mycobacterium chelonae</i> infection using whole genome sequencing and hybrid assembly

--Manuscript Draft--

**Manuscript Number:**	PONE-D-20-20588
**Article Type:**	Research Article
**Full Title:**	Investigating hospital <i>Mycobacterium chelonae</i> infection using whole genome sequencing and hybrid assembly
**Short Title:**	Investigating hospital <i>Mycobacterium chelonae</i> infection
**Corresponding Author:**	Frederic D Bushman<br>University of Pennsylvania<br>Philadelphia, Pennsylvania UNITED STATES
**Keywords:**	
**Abstract:**	<i>Mycobacterium chelonae </i> is a rapidly growing nontuberculous mycobacterium that is a common cause of nosocomial infections. Here we describe investigation of a possible nosocomial transmission of <i>M. chelonae</i> at the Hospital of the University of Pennsylvania (HUP). <i>M. chelonae</i> strains with similar high-level antibiotic resistance patterns were isolated from two patients who developed post-operative infections at HUP in 2017, suggesting a possible point source infection. The isolates, along with other clinical isolates from other patients, were sequenced using the Illumina and Oxford Nanopore technologies. The resulting short and long reads were hybrid assembled into draft genomes. The genomes were compared by quantifying single nucleotide variants in the core genome and assessed using a control dataset for identity. We show that that all <i>M. chelonae</i> isolates tested were highly divergent, consistent with environmental acquisition. Additionally, antibiotic resistance genes were predicted for our isolates, and several single nucleotide variants identified with the potential to modulated drug susceptibility, providing candidate resistance mechanisms.
**Order of Authors:**	CHRISTOPHER GU
	Frederic D. Bushman
	Chunyu Zhao
	Casey Hofstaedter
	Pablo Tebas
	Laurel Glaser
	Robert Baldassano
	Kyle Bittinger
	Lisa Mattei
**Additional Information:**	
**Question**	**Response**
**Financial Disclosure**<br><br>Enter a financial disclosure statement that describes the sources of funding for the work included in this submission. Review the submission guidelines for detailed requirements. View published research articles from *PLOS ONE* for specific examples.	This work was supported by the Penn Center for AIDS Research P30 AI 045008 (https://www.med.upenn.edu/cfar)under FDB; and the PennCHOP Microbiome Program (https://pennchopmicrobiome.chop.edu). The funders had no role in study design, data collection and analysis, decision to publish, or preparation of the manuscript.

Powered by Editorial Manager® and ProduXion Manager® from Aries Systems Corporation

This statement is required for submission and **will appear in the published article** if the submission is accepted. Please make sure it is accurate.<br><br>**Unfunded studies**<br>Enter: *The author(s) received no specific funding for this work.*<br><br>**Funded studies**<br>Enter a statement with the following details:<br>• Initials of the authors who received each award<br>• Grant numbers awarded to each author<br>• The full name of each funder<br>• URL of each funder website<br>• Did the sponsors or funders play any role in the study design, data collection and analysis, decision to publish, or preparation of the manuscript?<br>• **NO** - Include this sentence at the end of your statement: *The funders had no role in study design, data collection and analysis, decision to publish, or preparation of the manuscript.*<br>• **YES** - Specify the role(s) played.<br><br>* typeset	
**Competing Interests**<br><br>Use the instructions below to enter a competing interest statement for this submission. On behalf of all authors, disclose any competing interests that could be perceived to bias this work—acknowledging all financial support and any other relevant financial or non-financial competing interests.<br><br>This statement **will appear in the published article** if the submission is accepted. Please make sure it is accurate. View published research articles from *PLOS ONE* for specific examples.	The authors have declared that no competing interests exist.

**NO authors have competing interests**<br><br>Enter: *The authors have declared that no competing interests exist.*<br><br>**Authors with competing interests**<br><br>Enter competing interest details beginning with this statement:<br><br>*I have read the journal's policy and the authors of this manuscript have the following competing interests: [insert competing interests here]*<br><br>* typeset	
**Ethics Statement**<br><br>Enter an ethics statement for this submission. This statement is required if the study involved:<br><br>• Human participants<br>• Human specimens or tissue<br>• Vertebrate animals or cephalopods<br>• Vertebrate embryos or tissues<br>• Field research<br><br>Write "N/A" if the submission does not require an ethics statement.<br><br>General guidance is provided below. Consult the submission guidelines for detailed instructions. **Make sure that all information entered here is included in the Methods section of the manuscript.**	Penn IRB approved collection of human samples by the clinical Microbiology Laboratory at the Hospital of the University of Pennsylvania under the IRB protocol #829497.

Powered by Editorial Manager® and ProduXion Manager® from Aries Systems Corporation

**Format for specific study types**<br><br>**Human Subject Research (involving human participants and/or tissue)**<br>• Give the name of the institutional review board or ethics committee that approved the study<br>• Include the approval number and/or a statement indicating approval of this research<br>• Indicate the form of consent obtained (written/oral) or the reason that consent was not obtained (e.g. the data were analyzed anonymously)<br><br>**Animal Research (involving vertebrate animals, embryos or tissues)**<br>• Provide the name of the Institutional Animal Care and Use Committee (IACUC) or other relevant ethics board that reviewed the study protocol, and indicate whether they approved this research or granted a formal waiver of ethical approval<br>• Include an approval number if one was obtained<br>• If the study involved *non-human primates*, add *additional details* about animal welfare and steps taken to ameliorate suffering<br>• If anesthesia, euthanasia, or any kind of animal sacrifice is part of the study, include briefly which substances and/or methods were applied<br><br>**Field Research**<br><br>Include the following details if this study involves the collection of plant, animal, or other materials from a natural setting:<br>• Field permit number<br>• Name of the institution or relevant body that granted permission	
**Data Availability**<br><br>Authors are required to make all data underlying the findings described fully available, without restriction, and from the time of publication. PLOS allows rare exceptions to address legal and ethical concerns. See the PLOS Data Policy and FAQ for detailed information.	Yes - all data are fully available without restriction

A Data Availability Statement describing where the data can be found is required at submission. Your answers to this question constitute the Data Availability Statement and **will be published in the article**, if accepted.<br><br>**Important:** Stating 'data available on request from the author' is not sufficient. If your data are only available upon request, select 'No' for the first question and explain your exceptional situation in the text box.<br><br>Do the authors confirm that all data underlying the findings described in their manuscript are fully available without restriction?	
**Describe where the data may be found in full sentences. If you are copying our sample text, replace any instances of XXX with the appropriate details.**<br><br>• If the data are **held or will be held in a public repository**, include URLs, accession numbers or DOIs. If this information will only be available after acceptance, indicate this by ticking the box below. For example: *All XXX files are available from the XXX database (accession number(s) XXX, XXX.*).<br>• If the data are all contained **within the manuscript and/or Supporting Information files**, enter the following: *All relevant data are within the manuscript and its Supporting Information files.*<br>• If neither of these applies but you are able to provide **details of access elsewhere**, with or without limitations, please do so. For example:<br><br>*Data cannot be shared publicly because of [XXX]. Data are available from the XXX Institutional Data Access / Ethics Committee (contact via XXX) for researchers who meet the criteria for access to confidential data.*<br><br>*The data underlying the results presented in the study are available from (include the name of the third party*	Nanoflow is available at www.github.com/zhaoc1/nanflow while all other computer code used in this study is available at www.github.com/chrgu. M. chelonae and V. campbellii assembled genomes available at GenBank under project PRJNA594977.

and contact information or URL).<br>• This text is appropriate if the data are owned by a third party and authors do not have permission to share the data.<br><br>* typeset	
Additional data availability information:	Tick here if the URLs/accession numbers/DOIs will be available only after acceptance of the manuscript for publication so that we can ensure their inclusion before publication.

Powered by Editorial Manager® and ProduXion Manager® from Aries Systems Corporation

Manuscript
Click here to access/download;Manuscript;Manuscript.docx

Investigating hospital *Mycobacterium chelonae* infection using whole genome sequencing and hybrid assembly

Christopher H. Gu[1]*, Chunyu Zhao[2]*, Casey Hofstaedter[2], Pablo Tebas[3], Laurel Glaser[4], Robert Baldassano[2], Kyle Bittinger[2]+, Lisa M. Mattei[2]+, Frederic D. Bushman[1]+

1. University of Pennsylvania School of Medicine, Department of Microbiology, Philadelphia, PA, USA
2. Division of Gastroenterology, Hepatology, and Nutrition, Children's Hospital of Philadelphia, Philadelphia, PA, USA
3. University of Pennsylvania School of Medicine, Department of Medicine, Philadelphia, PA, USA
4. University of Pennsylvania School of Medicine, Department of Pathology and Laboratory Medicine, Philadelphia, PA, USA

*these authors contributed equally

+ corresponding authors

Author contributions:

Conceptualization: CHG, CZ, PT, LG, LMM, KB, RB, FDB

Data curation, formal analysis, software, validation, visualization: CHG, CZ

Investigation, methodology: CHG, CZ, CH, LG, LMM

Resources: CHG, CZ, CH, PT, LG, LMM, FDB

Supervision, funding acquisition, project administration: RB, KB, FDB

Writing – Original draft preparation: CHG, FDB

Writing – Review & Editing: CHG, CZ, CH, PT, LG, RB, KB, LMM, FDB

Corresponding authors information:

Frederic D. Bushman

Department of Microbiology

3610 Hamilton Walk

426A Johnson Pavilion

Philadelphia, PA 19140-6076

bushman@pennmedicine.upenn.edu

(215) 573-8732

https://orcid.org/0000-0003-4740-4056

Lisa Mattei

Leonard and Madlyn Abramson Pediatric Research Center

3615 Civic Center Blvd, 9th floor

Philadelphia, PA 19104-6076

matteil@email.chop.edu

https://orcid.org/0000-0002-2238-0281

Kyle Bittinger

The Children's Hospital of Philadelphia

Microbiome Center

Roberts Center of Pediatric Research

2716 South Street, 14th floor

Philadelphia, PA 19146-2305

bittingerk@email.chop.edu
(267) 425-1641
https://orcid.org/0000-0003-3472-5934

Abstract – 159

Mycobacterium chelonae is a rapidly growing nontuberculous mycobacterium that is a common cause of nosocomial infections. Here we describe investigation of a possible nosocomial transmission of *M. chelonae* at the Hospital of the University of Pennsylvania (HUP). *M. chelonae* strains with similar high-level antibiotic resistance patterns were isolated from two patients who developed post-operative infections at HUP in 2017, suggesting a possible point source infection. The isolates, along with other clinical isolates from other patients, were sequenced using the Illumina and Oxford Nanopore technologies. The resulting short and long reads were hybrid assembled into draft genomes. The genomes were compared by quantifying single nucleotide variants in the core genome and assessed using a control dataset for identity. We show tha[illegible]at all *M. chelonae* isolates tested were highly divergent[illegible]nsistent with environmental acquisition. Additionally, antibiotic resistance genes were predicted for our isolates, and several single nucleotide variants identified with the potential to modulated drug susceptibility, providing candidate resistance mechanisms.

Text – 3,699

Introduction

Mycobacterium chelonae, a rapidly growing nontuberculous mycobacterium (NTM), is ubiquitous in the environment and is a common source of infection in humans. *M. chelonae* has caused several outbreaks from single point sources, but no human-to-human transmission has been observed to date (1–6). *M. chelonae* is most commonly associated with nosocomial soft tissue infections of the skin and eye, but can also cause catheter-associated infections, disseminated and invasive infections, and pulmonary infections (1). *Mycobacterium* can be highly resistant to many antibiotics because of their naturally impermeable cell walls as well as mutation of bacterial genes that encode antibiotic targets (7–9).

Among the *M. chelonae* isolates collected in 2017 from the Hospital of the University of Pennsylvania (HUP) clinical microbiology laboratory were two isolates from female patients who developed post-operative surgical site infections in breast tissue. The patients underwent surgery within the same year, and yielded isolates showing similar resistance patterns, raising the possibly of a nosocomial point source infection. To investigate this possibility, we performed whole genome sequencing on every *M. chelonae* isolate collected by the HUP clinical microbiology laboratory over a one-year period (n=7) and examined differences in the single nucleotide variants (SNVs) of each isolate's core genome. As a control, we created a dataset from a single *Vibrio campbellii* strain that was sequenced 39 times independently, allowing us to determine a threshold of SNVs that would distinguish different strains from sequencing error. Finally, we investigated genes and SNVs related to drug resistance in each isolate.

Methods

Samples were collected as part of routine clinical practice with patient consent and sent to the Clinical Microbiology Laboratory at the Hospital of the University of Pennsylvania under the IRB protocol #829497. Respiratory samples were decontaminated with NaOH and N-acetylcysteine and tissue specimens were pulverized in a tissue grinder. Prepared specimens were inoculated on 7H11 selective and non-selective solid media and a Mycobacterial Growth Indicator Tube (MGIT) broth. All cultures were incubated at 35-37°C for 6 weeks. Positive cultures for *Mycobacteria* were identified at the species level using *hsp65* gene sequencing. Susceptibility testing and MIC determination was performed using the RAPMYCO microbroth dilution plate (ThermoFisher) and susceptibility was determined using the CLSI M42 A2. Tigecycline susceptibility breakpoints for the MIC have not been established for mycobacterium (10). For our analysis, we based our tigecycline thresholds for resistance based on the Wallace et al. (11). For antibiotic resistance gene investigation, we categorized susceptibility into susceptible, intermediate, and resistant.

Mycobacterium samples had been previously frozen for routine clinical purposes and were re-isolated on chocolate agar and propagated by growth in Middlebrook 7H9 media for 5 days. Multiple DNA purification methods were compared to identify one producing high molecular weight DNA in good yield. Ultimately, DNA was purified from each sample using a phenol-chloroform DNA extraction designed for high molecular weight DNA (12). Long-read libraries were prepared using the Rapid Barcoding Kit, version SQK-RBK004 (Oxford Nanopore, Oxford, UK) and sequenced on the MinION using a R9.4.1 flow cell. Short-read libraries were prepared using the TruSeq

DNA Nano Library Prep Kit (Illumina, San Diego, CA), and sequenced on the HiSeq 2500 using 2x125 bp chemistry. *V. campbellii* was grown in Difco Marine broth 2216 culture media (BD) overnight and DNA was extracted using DNeasy Blood & Tissue Kits (Qiagen). Short read libraries were prepared using the Nextera XT Library Prep Kit (Illumina, San Diego, CA) and sequenced on the HiSeq 2500 using 2x125bp chemistry. References genomes were all isolates that were whole genome sequenced and collected from GenBank.

We used the Sunbeam pipeline to process the short reads and an in-house pipeline, Nanoflow (https://github.com/zhoac1/nanoflow), to process the long reads and perform the hybrid assembly (13). Short read processing included trimming adapters off reads, filtering out low quality reads and removing low complexity reads (13). Long read processing included base calling using Albacore, trimming adaptors, and filtering and subsetting reads based on quality using Porechop (https://github.com/rrwick/Porechop) and Filtlong (https://github.com/rrwick/Filtlong). Hybrid assembly was performed by two methods: 1) using Canu, polishing with Nanopolish and correction by short reads with Pilon or 2) using Unicycler, a program that uses the short read assembler Spades guided by long reads as scaffolds, which is further polished by Pilon (14,15). CheckM and alignment to reference genomes were used to check the quality of the draft genomes (16).

We used another in-house pipeline, CoreSNPs (https://github.com/chrgu/coreSNPs) to investigate how related the isolates were to each other. CoreSNPs uses Prokka for genome annotation, and Roary for investigating the pangenome and creating a hierarchical cluster based on the presence and absence of accessory genes (17,18). An in-house R script and shell script were used to extract the

core genes from the isolates to compare SNVs by hamming distance. The SNV analysis used SamTools and SNP-sites (19,20). Approximately maximum-likelihood phylogenetic trees for the core genes were generated by Fasttree 2 (21).

The threshold for identity was determined using a dataset of a single isolate of *V. campbellii* sequenced 39 times (Illumina only) and examining the number of SNVs between sequencing runs. The dataset was analyzed using the same in-house pipelines as above. Roary defined core genes as genes present in 100% of the samples; 643 genes, or ~12.5%, were not 100% conserved, likely due to gaps between contigs in the short read only draft genomes.

We used Resistance Gene Identifier (RGI) on the output of Prokka to examine resistance genes within the genome (22). We searched for both high identity and low identity homologous hits to identify previously known and potentially novel resistance genes, respectively. We also manually aligned known genes that can harbor resistance mutations with Muscle to identify and compare mutations within the gene that corresponded with drug susceptibility (23).

Nanoflow is available at www.github.com/zhaoc1/nanflow while all other computer code used in this study is available at www.github.com/chrgu. *M. chelonae* and *V. campbellii* assembled genomes available at GenBank under project PRJNA594977.

Results

M. chelonae was isolated from seven patients at the Hospital of the University of Pennsylvania in 2017 (Table 1), including the two breast tissue cases. Ages ranged from

46 to 64. Sites of infection included skin (n=2), breast tissue (n=2), and respiratory tract (sampled as bronchial alveolar lavage (n=1) and sputum (n=2))

Table 1. Patient demographics and suspected pair of transmission.

ID	SUSPECTED NOSOCOMIAL INFECTION PAIR*	AGE	SEX	SITE OF COLLECTION
**MYCO1**	X	45	Female	breast
**MYCO2**	X	47	Female	breast
**MYCO3**		61	Female	skin
**MYCO4**		64	Male	bronchiolar lavage
**MYCO5**		61	Male	sputum
**MYCO6**		61	Male	sputum
**MYCO7**		55	Male	leg skin

Drug susceptibility testing was performed on each isolate with 11 different antibiotics to determine the minimum inhibitory concentration (MIC) (Table 2). As expected, most of the isolates of *M. chelonae* were highly resistant to antibiotics, with only a few drugs such as clarithromycin, tigecycline, and tobramycin, effective against all the strains.

Table 2. Antibiotic resistance profile of M. chelonae isolates with 11 antibiotics.

ID	TMP[*]	LINE[†]	CIPRO[§]	IMI[¶]	MOXI[#]	CEFOX[**]	DOXY[††]	MINO[§§]	TIGE[¶¶]	TOBRA[##]	CLAR[***]
**MYCO1**	>8/152 (R)	32 (R)	4 (R)	16 (I)	8 (R)	>128 (R)	>16 (R)	4 (I)	0.5 (S)	4 (I)	0.5 (S)
**MYCO2**	>8/152 (R)	32 (R)	>4 (R)	>64 (R)	8 (R)	>128 (R)	>16 (R)	>8 (R)	0.5 (S)	2 (S)	0.5 (S)
**MYCO3**	>8/152 (R)	32 (R)	>4 (R)	16 (I)	8 (R)	128 (R)	>16 (R)	>8 (R)	0.5 (S)	2 (S)	0.5 (S)
**MYCO4**	4/76 (R)	8 (S)	4 (R)	16 (I)	4 (R)	>128 (R)	>16 (R)	>8 (R)	0.5 (S)	<1 (S)	0.25 (S)
**MYCO5**	8/152 (R)	16 (I)	4 (R)	32 (R)	8 (R)	>128 (R)	>16 (R)	>8 (R)	0.25 (S)	2 (S)	0.5 (S)
**MYCO6**	>8/152 (R)	16 (I)	2 (I)	32 (R)	4 (R)	64 (I)	1 (S)	2 (I)	1 (S)	4 (I)	0.5 (S)
**MYCO7**	>8/152 (R)	>32 (R)	2 (I)	32 (R)	4 (R)	>128 (R)	>16 (R)	>8 (R)	0.5 (S)	2 (S)	2 (S)

Footnote: [*]TMP-SMX, [†]Linezolid, [§]Ciprofloxacin, [¶]Imipenem, [#]Moxifloxacin, [**]Cefoxitin, [††]Doxycycline, [§§]Minocycline, [¶¶]Tigecycline, [##]Tobramycin, ***Clarithromycin

The *M. chelonae* strains were isolated from seven patients at HUP during routine clinical treatment. Frozen stocks of the *M. chelonae* isolates were cultured and DNA was extracted. Extensive optimization was required to allow lysis of the tough *Mycobacterium* cell wall while preserving long DNA chains (see methods). DNA sequencing data was acquired using the Illumina HiSeq 2500 to generate short reads and the Oxford Nanopore MinION to generate long reads. Long-read assembly was carried out by Canu and polished by Nanopolish. The long-read assemblies were either 1) corrected with short-reads by Pilon or 2) used as scaffolds to guide short-read assembly by Unicycler, then further polished by Pilon in our hybrid assembly pipeline. Both assembly methods were used with each isolate and the best draft genome was chosen based on completeness (by checkM), number and length of contigs, and alignment to a reference genome. For one of

our seven strains, Myco5, only short read assembly was carried out, due to difficulty in purifying high molecular weight DNA from this isolate.

In one case, two isolates were cultured from the same patient and analyzed separately (Myco3a/3b). In another case, a single genomic DNA preparation was sequenced and assembled twice (Myco2a/b). Both pairs provide further empirical data on the sources of error in library preparation and DNA sequencing.

The whole genome sequencing resulted in a range of contig numbers (n=1 to 76) comprising the main chromosome. For those that were hybrid assembled, the range of contigs was one to four. Three of the nine assemblies yielded complete circular contigs for the main chromosome. The genomes ranged in size from 4.95 to 5.20 Mbp. No clearly defined episomes were found, as judged by detection of extrachromosomal circles (Table S1).

We assessed the phylogenetic relationships by comparing the number of single nucleotide variants (SNVs) between cores genes (genes found in every isolate), which allowed us to interrogate potential transmission chains. We used all 43 whole genome sequences of *M. chelonae* present in GenBank as reference to construct an approximately maximum-likelihood phylogenetic tree. CheckM analysis were performed to ensure completion and quality of the reference genomes prior to analysis. Analysis of our set of *M. chelonae* genomes returned a total of 17,582 genes in the pan-genome, of which only 3,368 were considered core genes. The length of the total concatenated core genes per genome was 3,296,947 bases. Within the core genes, the number of SNVs between unique isolates ranged from 3,383 to 62,854. Our two samples from the same individual (Myco3a and Myco3b) differed by 3 SNVs while our technical replicates (Myco2a and

Myco2b) differed by 2 SNVs. The potential transmission pair, Myco1 and Myco2a/b differed by 16544/16542 SNVs in the core genes (SNVs are indicated for Myco2 replicates a and b, respectively). A maximum likelihood phylogenetic tree based on the SNVs data is shown in Fig 1A. There was no obvious clustering of isolates with database samples isolated from the same human body site, animal, or environmental source. Likewise, the tree based on presence or absence of accessory genes (Fig 1B) also showed the same.

Fig 1. Relationship of *M. chelonae* genome sequences.

A. An approximately maximum-likelihood phylogenetic tree showing relationships among *M. chelonae* isolates based on SNVs in the core genes. Isolates from our study are indicated with "Myco" and the isolate number. The sampling site and host of the isolate is coded by color and shape, respectively, at branch tips. The scale at the bottom represents the number of substitutions per sequence site based on length of the tree. B: An approximately-maximum-likelihood tree showing relationship among M. chelonae isolates based on presence or absence of accessory genes. C: SNVs per Mb core genome between *M. chelonae* isolates. SNVs, calculated as hamming distance between the core genes of all isolates divided by the total length of core genes. Myco2a/2b and Myco3a/3b are technical and biological replicates, respectively.

To assess the likelihood of infection from a common point source, we next empirically assessed the numbers of SNVs expected due to sequencing error in a larger set of genomes. As a positive control in shotgun metagenomic studies, we repeatedly

sequenced a single bacterium, *V. campbellii*, a luciferase-encoding marine bacterium, that was divergent from strains likely present in our samples. We recovered an average of 9,072,182 reads over 39 replicates, allowing generation of 39 full genome sequences. Analysis of the *V. campbellii* genomes using Roary disclosed 4495 core genes in our samples. Within the core genes, we found a range of SNVs from 0 to 74, with mean of 15 SNVs (Fig 2A). The total length for the concatenated core genes was 4,209,934 bases. The two most divergent *V. campbellii* assemblies also had low sequence coverage (Supplementary Table S2), indicating a rough upper bound on the number of SNV errors associated with suboptimal sequence acquisition.

Fig 2. Comparison of *Vibrio campbellii* genomes by SNVs per Mb core genome to develop statistics for calling identity.

A. The set of SNVs per Mb core genome, calculated by hamming distances divided by length of core genome between the sequence sets for identical DNAs. B. Graph showing the Hamming distances (x-axis) and the frequencies of distances between pairs (y-axis). The distances between the technical and biological replicates are marked (Myco2a and 2b, and Myco 3a and 3b), as is the distance between the candidate transmission pair (Myco1 and 2a).

This comparison takes advantage of Illumina sequence reads only, whereas our *M. chelonae* isolates were sequenced using hybrid assembly of short and long reads. We thus generated short read only assemblies for the *M. chelonae* isolates for a more direct comparison to the *V. campbellii* dataset. Our short-read-only genomes contained slightly

fewer core genes (3,143 vs 3,368) compared to our hybrid assemblies. The short-read genomes also had more SNVs per Mbp core genes. Both of our technical replicates showed slightly higher SNV counts (3 and 64), but were lower than the maximum number of SNVs for identical strains in our control *V. campbellii* dataset (the maximum number of SNVs for any pair of isolates was 74).

The possible transmission pair, isolates Myco1 and Myco2a/b (Fig 3), differed by 16,542/16,544 SNVs in the core genes (Fig 2B), providing strong evidence that they are different strains and not related by direct person-to-person transmission. This corresponds to a difference of 5,237.58 SNVs per Mbp core genes. The smallest difference between our isolated *M. chelonae* strains was 3426 SNVs in the core genes or 1038.18 SNVs per Mbp core genes. Our technical replicates Myco3a and Myco3b differed by 3 SNVs (0.91 SNVs per Mbp core genes), and Myco2a and Myco2b differed by 2 SNVs (0.61 SNVs per Mbp core genes) (Fig 1C). For comparison, the mean number of SNVs in pairwise comparisons of *V. campbelli* control assemblies was 5.71 SNVs per Mbp core genes; the maximum number was 17.62 SNVs per Mbp core genes (Fig 2B). The number of SNVs in the candidate transmission pair thus far exceeds the number of SNVs that could be generated by sequencing error per Mbp as seen from the *V. campbellii* controls and exceeds the SNVs generated in our *M. chelonae* replicates (p-value < 0.001 by binomial test). Thus, the data do not support transmission of a common strain from a single point source, or a transmission event between the patients (Fig 2B).

Fig 3. Comparison of genomes from the candidate transmission pair.

Circos plots are shown of assemblies of Myco1 (left) and Myco2b (right). Each ring represents, from inner to outer ring GC content; GC skew; RNA genes; genes unique to the isolate, colored by COG function; and genes shared between the two isolates, colored by COG function. Each genome was rotated to the origin of replication at the top.

The molecular determinants of antibiotic resistance in *M. chelonae* are not well studied, so we sought to assess possible mechanisms disclosed in our sequence data. Analysis using Resistance Gene Identifier (RGI) searching for high identity hits to previously known genes related to resistance (22), yielded two partial hits to genes in all the isolates, and a third in three of seven isolates. The two partial hits in all isolates were *LRA-3* (100% identity) and *erm(38)* (80% identity); the third gene was *mtrA* (95.17% identity). *LRA-3* is a gene coding for a beta-lactamase originally identified in soil samples and could possibly contribute imipenem resistance (24). The gene, *erm(38)*, encodes for a 23S dimethyltransferase found in *Mycobacterium smegmatis* and can provide resistance to macrolides and lincosomides (25). It has been shown to increase resistance to Clarithromycin (26). *mtrA* is a gene from *Mycobacterium tuberculosis* whose expression has been shown to influence cell morphology and drug resistance in *Mycobacterium smegmatis* (27,28).

We also used RGI to identify genes with lower homology to known resistance genes, providing possible starting points for follow up studies. We found a list of candidate genes that may contribute to resistance and further narrowed down the list using the susceptibility testing data by filtering out genes that were present in susceptible populations and genes that were not present in intermediate or fully resistant populations.

Only 6 of the 11 drugs had low identity matches associated with their resistance pattern: TMP-SMX (sulfonamide), ciprofloxacin (fluoroquinolone), moxifloxacin (fluoroquinolone), imipenem (carbapenem), cefoxitin (cephalosporin), and Minocycline (tetracycline). Genes are listed in Supplementary Table S3.

We further examined genes reported to be targets of mutations that may cause resistance, such as mutations in 23S rRNA for linezolid resistance and *gyrA* for fluoroquinolone resistance. There were no known or novel mutations present in the 23S rRNA genes in our isolates that correlated with drug susceptibility or resistance. For *gyrA*, we compared the two intermediate resistant isolates (Myco6 and Myco7) to five resistant isolates. We found that there were no consistent mutations that resulted in resistance, but Myco6 had two mutations (S710R and E773D) not present in the other isolates and Myco7 had one mutation not present in the other isolates (T832A). These amino acid changes might potentially modulate th[illegible]esistance phenotype, though functional confirmation is needed.

Discussion

Here we investigated the possibility of a nosocomial *M. chelonae* transmission at the HUP in 2017 and found that [illegible] there is no evidence to support a point source outbreak. Our analysis found each isolate of *M. chelonae* was no more similar to each other than to the strains collected from the NCBI database. This further supports that these infections are likely acquired from the environment and not from a single point source. Our analysis also supports the use of next generation sequencing to assess possible nosocomial transmissions within the hospital environment.

To provide a control for sequencing error, we developed a dataset to help set a threshold for use in calling identity between two isolates that takes account of error in DNA sequence determination. We sequenced a strain of *V. campbellii* using the Illumina method as a control 39 times independently. We found that the depth of sequencing coverage correlated with the number of SNVs found in the genome assembly. The number of SNVs ranged from 0 to 74 SNVs depending on which pair of sequencing runs was compared, despite the same input DNA. There were several runs that though following the same protocol, had higher numbers of SNVs on average when compared to sequencing results of other runs, and these correlated with low sequence coverage (Supplementary Table S2). Going forward, the data presented here provides a threshold for comparing microbial genomes generally.

Our analysis also included two samples collected from the same individual and a single isolate sequenced twice, again to help evaluate thresholds for calling identity despite sequencing error. We found that our two samples from the same patient only differed by 0.91 SNVs per Mbp core genes after our hybrid assembly; our single isolate sequenced twice differed by 0.61 SNVs per Mbp core genes. These control *M. chelonae* genomes show SNV numbers well within the range expected for identical sequences based on our *V. campbellii* dataset. This indicates that the sequencing and hybrid assembly pipeline works as expected to generate high quality genomes and allows identification of identical organisms sequenced separately.

Our analysis also shed light on the host preferences of sequenced *M. chelonae* strains. Some of our isolates were related to environmental samples such as water and sea

creatures, while others clustered with human isolates. Our data did not provide evidence for a strongly human-associated clade.

Our investigation of the genetic basis of *M. chelonae* drug resistance was inconclusive. Examination with Resistant Gene Identifier provided us a list with possible resistance genes. While there were a few high identity resistance gene hits in our isolates, the presence of these genes did not correspond with the drug susceptibility phenotypes. For example, every isolate had an 80% identity hit to *erm(38)*, a gene found in *M. smegmatis* to mediate resistance to macrolides, such as clarithromycin, but all of the isolates were susceptible to clarithromycin. Thus, it is possible that the *erm(38)* homolog in *M. chelonae* performs a different role. For *LRA-3*, a metallo-beta-lactamase gene, all isolates were intermediately or fully resistant to the carbapenem tested. Since there are two phenotypes in the presence of the gene, this suggests that it may play a role in resistance, but other mechanisms may be involved as well. We also assessed low homology hits to resistance genes and mutations in genes that are known to influence drug susceptibility. For both, we have a narrow list of possible genes or mutations, but given our low sample size, it is likely that most are false positives, and all require further validation to confirm. Resistance in *M. chelonae* may in part be due to blocking entry of antibiotics by the tough cell wall documented previously (29). Data presented here may help guide future studies of mechanisms of resistance in *M. chelonae*.

In conclusion, we investigated a possible nosocomial outbreak of *M. chelonae* at HUP. Our analysis concluded that no point source transmission occurred and that each case of *M. chelonae* involved clearly distinct strains, likely acquired from the environment. Our analysis also includes a dataset to help determine thresholds for

evaluating identity between different strains while controlling for sequencing error. Finally, we queried potential antibiotic resistance mechanisms by genomic analysis, providing candidate genes and mutations for potential follow up.

Acknowledgements

We are grateful to members of the Bushman laboratory for help and suggestions; and Laurie Zimmerman for help with figures.

Financial Disclosure Statement

This work was supported by the Penn Center for AIDS Research P30 AI 045008; and the PennCHOP Microbiome Program (https://pennchopmicrobiome.chop.edu).

References

1. Akram SM, Saleh D. Mycobacterium Chelonae. In Treasure Island (FL); 2019.
2. Donohue MJ, Wymer L. Increasing prevalence rate of nontuberculous mycobacteria infections in five states, 2008-2013. Ann Am Thorac Soc. 2016;
3. Wang HX, Yue J, Han M, Yang JH, Gao RL, Jing LJ, et al. Nontuberculous mycobacteria: Susceptibility pattern and prevalence rate in Shanghai from 2005 to 2008. Chin Med J (Engl). 2010;
4. Kennedy BS, Bedard B, Younge M, Tuttle D, Ammerman E, Ricci J, et al. Outbreak of Mycobacterium chelonae Infection Associated with Tattoo Ink . N Engl J Med. 2012;
5. Meyers H, Brown- Elliott BA, Moore D, Curry J, Truong C, Zhang Y, et al. An

Outbreak of Mycobacterium chelonae Infection Following Liposuction . Clin Infect Dis. 2002;

6. Freitas D, Alvarenga L, Sampaio J, Mannis M, Sato E, Sousa L, et al. An outbreak of Mycobacterium chelonae infection after LASIK. Ophthalmology. 2003;

7. Monego F, Duarte RS, Biondo AW. gyrA and gyrB Gene Mutation in Ciprofloxacin-Resistant Mycobacterium massiliense Clinical Isolates from Southern Brazil. Microb Drug Resist [Internet]. 2012;18(1):1–6. Available from: https://doi.org/10.1089/mdr.2011.0047

8. Hillemann D, Rüsch-Gerdes S, Richter E. In vitro-selected linezolid-resistant Mycobacterium tuberculosis mutants [4]. Antimicrobial Agents and Chemotherapy. 2008.

9. Nessar R, Reyrat JM, Murray A, Gicquel B. Genetic analysis of new 16s rRNA mutations conferring aminoglycoside resistance in Mycobacterium abscessus. J Antimicrob Chemother. 2011;

10. Hatakeyama S, Ohama Y, Okazaki M, Nukui Y, Moriya K. Antimicrobial susceptibility testing of rapidly growing mycobacteria isolated in Japan. BMC Infect Dis. 2017;

11. Wallace RJ, Brown-Elliott BA, Crist CJ, Mann L, Wilson RW. Comparison of the in vitro activity of the glycylcycline tigecycline (formerly GAR-936) with those of tetracycline, minocycline, and doxycycline against isolates of nontuberculous mycobacteria. Antimicrob Agents Chemother. 2002;

12. Van Soolingen D, Hermans PWM, De Haas PEW, Soll DR, Van Embden JDA. Occurrence and stability of insertion sequences in Mycobacterium tuberculosis

complex strains: Evaluation of an insertion sequence-dependent DNA polymorphism as a tool in the epidemiology of tuberculosis. J Clin Microbiol. 1991;

13. Clarke EL, Taylor LJ, Zhao C, Connell A, Lee JJ, Fett B, et al. Sunbeam: An extensible pipeline for analyzing metagenomic sequencing experiments. Microbiome. 2019;
14. Wick RR, Judd LM, Gorrie CL, Holt KE. Unicycler: Resolving bacterial genome assemblies from short and long sequencing reads. PLoS Comput Biol. 2017;13(6):1–22.
15. Bankevich A, Nurk S, Antipov D, Gurevich AA, Dvorkin M, Kulikov AS, et al. SPAdes: A New Genome Assembly Algorithm and Its Applications to Single-Cell Sequencing. J Comput Biol. 2012;
16. Parks DH, Imelfort M, Skennerton CT, Hugenholtz P, Tyson GW. CheckM: Assessing the quality of microbial genomes recovered from isolates, single cells, and metagenomes. Genome Res. 2015;
17. Seemann T. Prokka: Rapid prokaryotic genome annotation. Bioinformatics. 2014;
18. Page AJ, Cummins CA, Hunt M, Wong VK, Reuter S, Holden MTG, et al. Roary: Rapid large-scale prokaryote pan genome analysis. Bioinformatics. 2015;
19. Li H, Handsaker B, Wysoker A, Fennell T, Ruan J, Homer N, et al. The Sequence Alignment/Map format and SAMtools. Bioinformatics. 2009;
20. Page AJ, Taylor B, Delaney AJ, Soares J, Seemann T, Keane JA, et al. SNP-sites: rapid efficient extraction of SNPs from multi-FASTA alignments. Microb Genomics. 2016;

21. Price MN, Dehal PS, Arkin AP. FastTree 2 - Approximately maximum-likelihood trees for large alignments. PLoS One. 2010;

22. Jia B, Raphenya AR, Alcock B, Waglechner N, Guo P, Tsang KK, et al. CARD 2017: Expansion and model-centric curation of the comprehensive antibiotic resistance database. Nucleic Acids Res. 2017;

23. Edgar RC. MUSCLE: Multiple sequence alignment with high accuracy and high throughput. Nucleic Acids Res. 2004;

24. Allen HK, Moe LA, Rodbumrer J, Gaarder A, Handelsman J. Functional metagenomics reveals diverse B-lactamases in a remote Alaskan soil. ISME J. 2009;

25. Madsen CT, Jakobsen L, Douthwaite S. Mycobacterium smegmatis Erm(38) is a reluctant dimethyltransferase. Antimicrob Agents Chemother. 2005;

26. Nash KA. Intrinsic macrolide resistance in Mycobacterium smegmatis is conferred by a novel erm gene, erm(38). Antimicrob Agents Chemother. 2003;

27. Rouquette C, Harmon JB, Shafer WM. Induction of the mtrCDE-encoded efflux pump system of Neisseria gonorrhoeae requires MtrA, an AraC-like protein. Mol Microbiol. 1999;

28. Li Y, Zeng J, Zhang H, He ZG. The characterization of conserved binding motifs and potential target genes for M. tuberculosis MtrAB reveals a link between the two-component system and the drug resistance of M. smegmatis. BMC Microbiol. 2010;

29. Jarlier V, Nikaido H. Mycobacterial cell wall: Structure and role in natural resistance to antibiotics. FEMS Microbiology Letters. 1994.

Supplementary Material

S1 Table. Genome sequences analyzed in this study.

S2 Table. ***Vibrio campbellii*** **assembly information.**

S3 Table. List of potential ***Mycobacterium chelonae*** **resistance genes by drug type**

Figure 1a Click here to access/download;Figure;Fig1a.tif

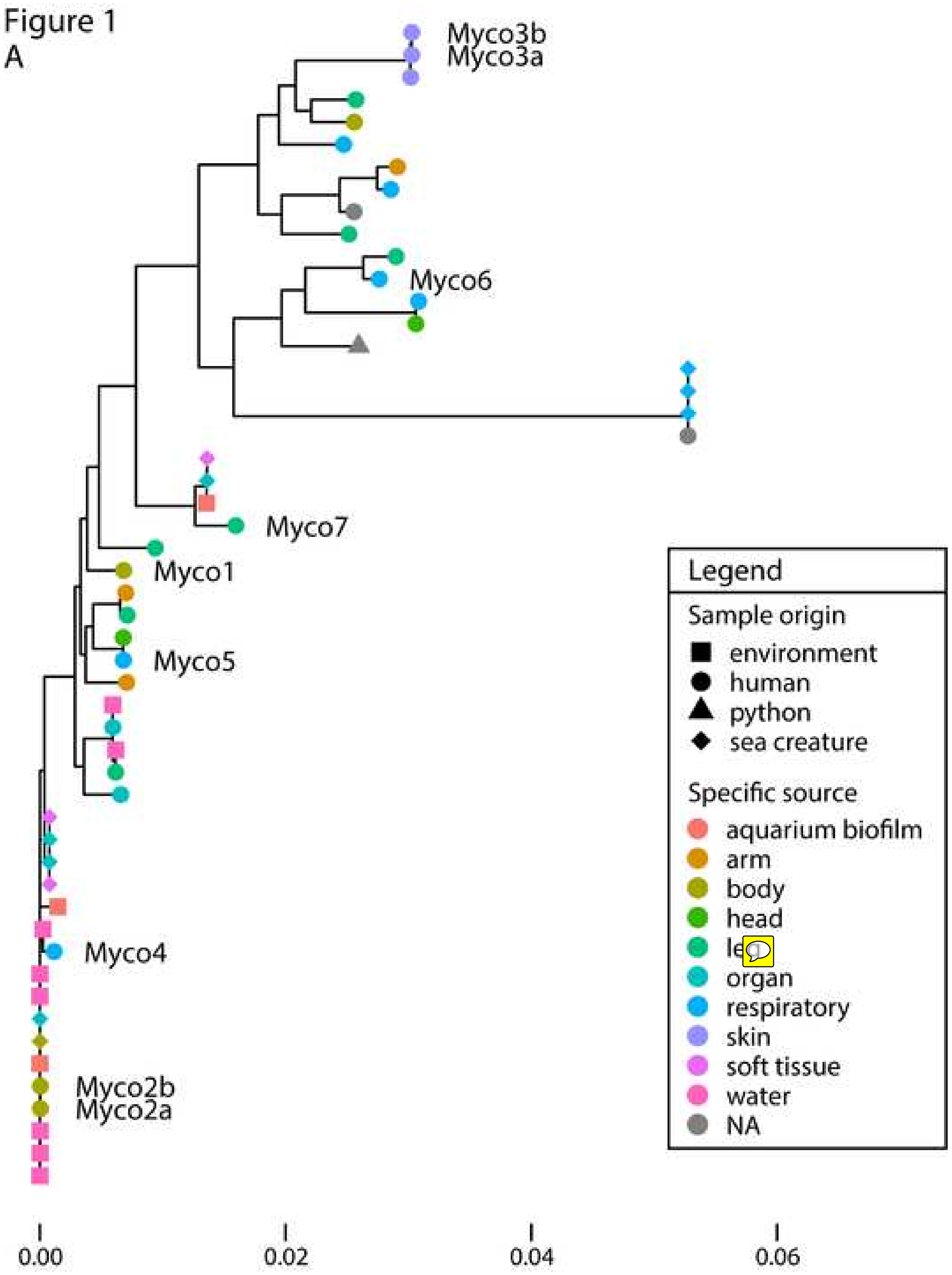

Figure 1b

Click here to access/download;Figure;Fig1b.tif

Figure 1
B

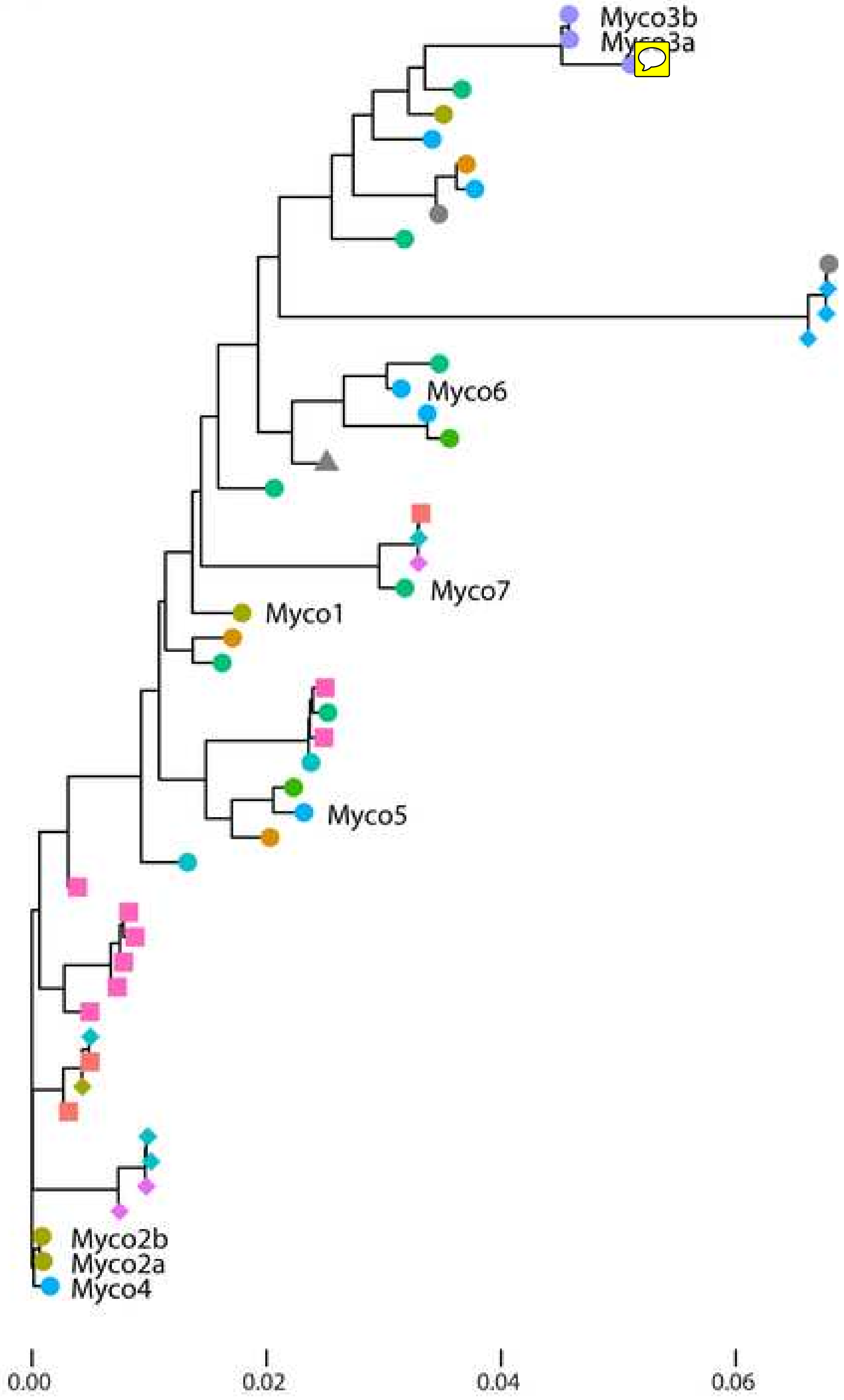

 Click here to access/download;Figure;Fig1c.tif

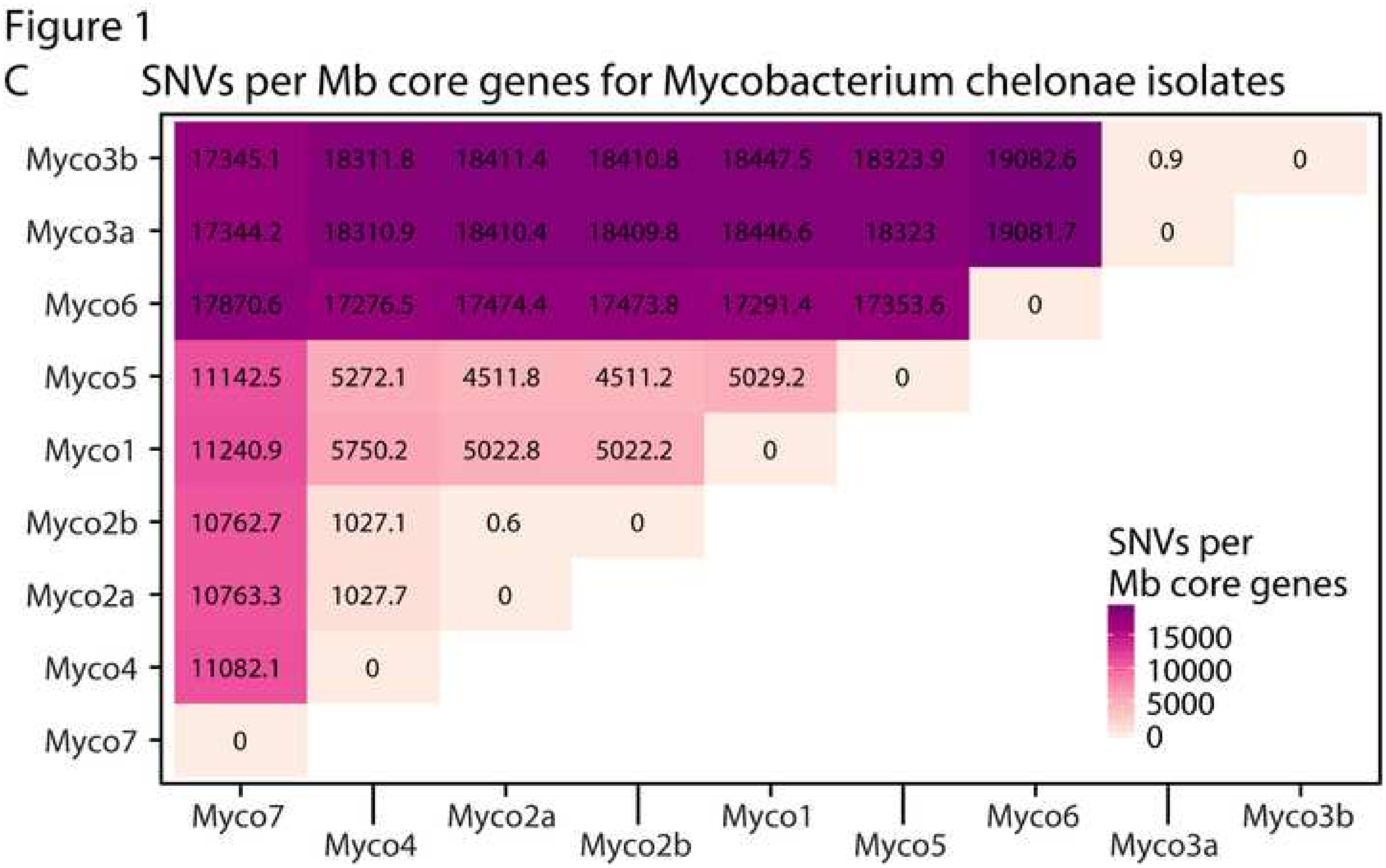

Figure 2a Click here to access/download;Figure;Fig2a.tif

Figure 2
A

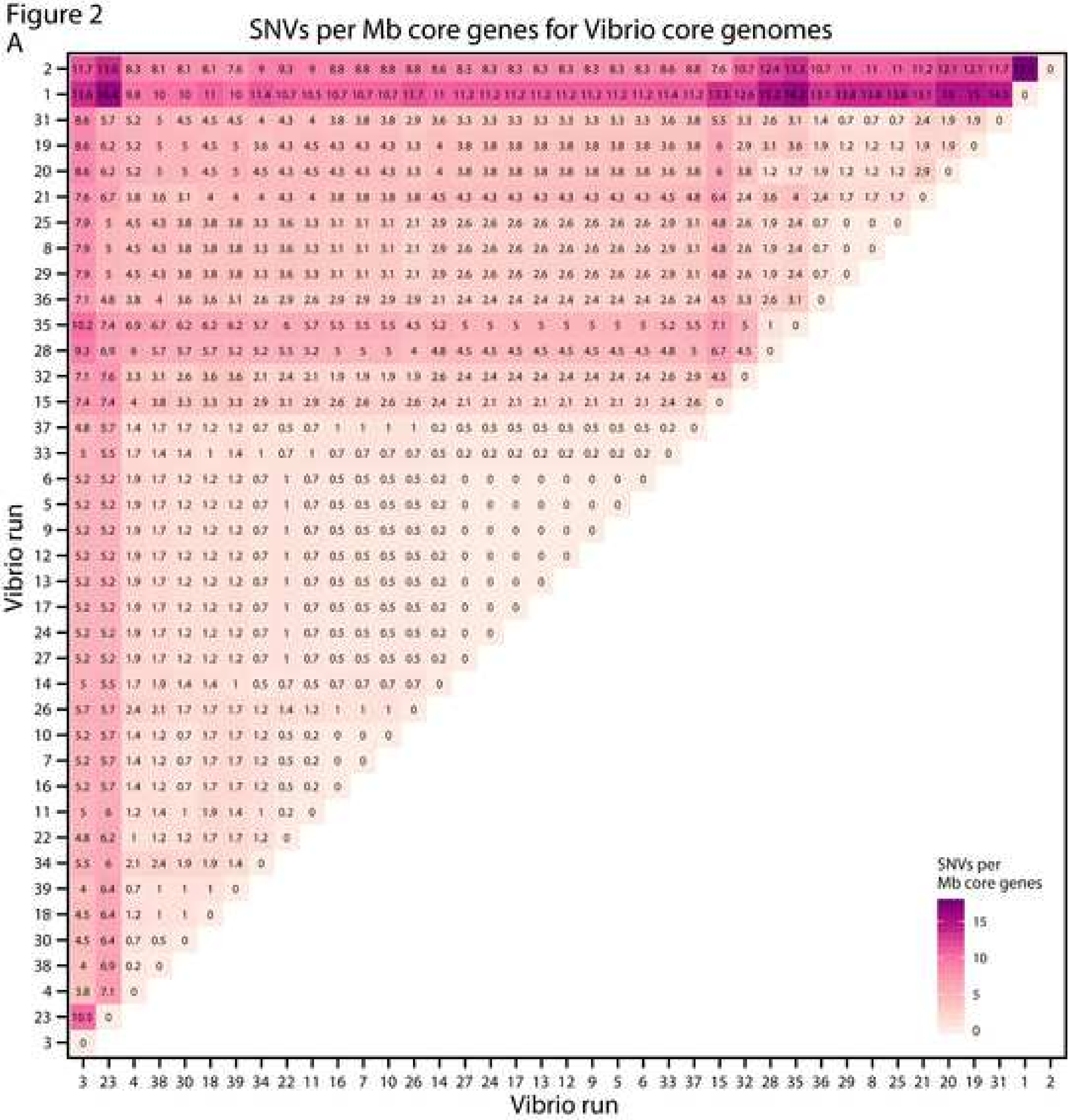

Figure 2b

Click here to access/download;Figure;Fig2b.tif

Figure 2

B

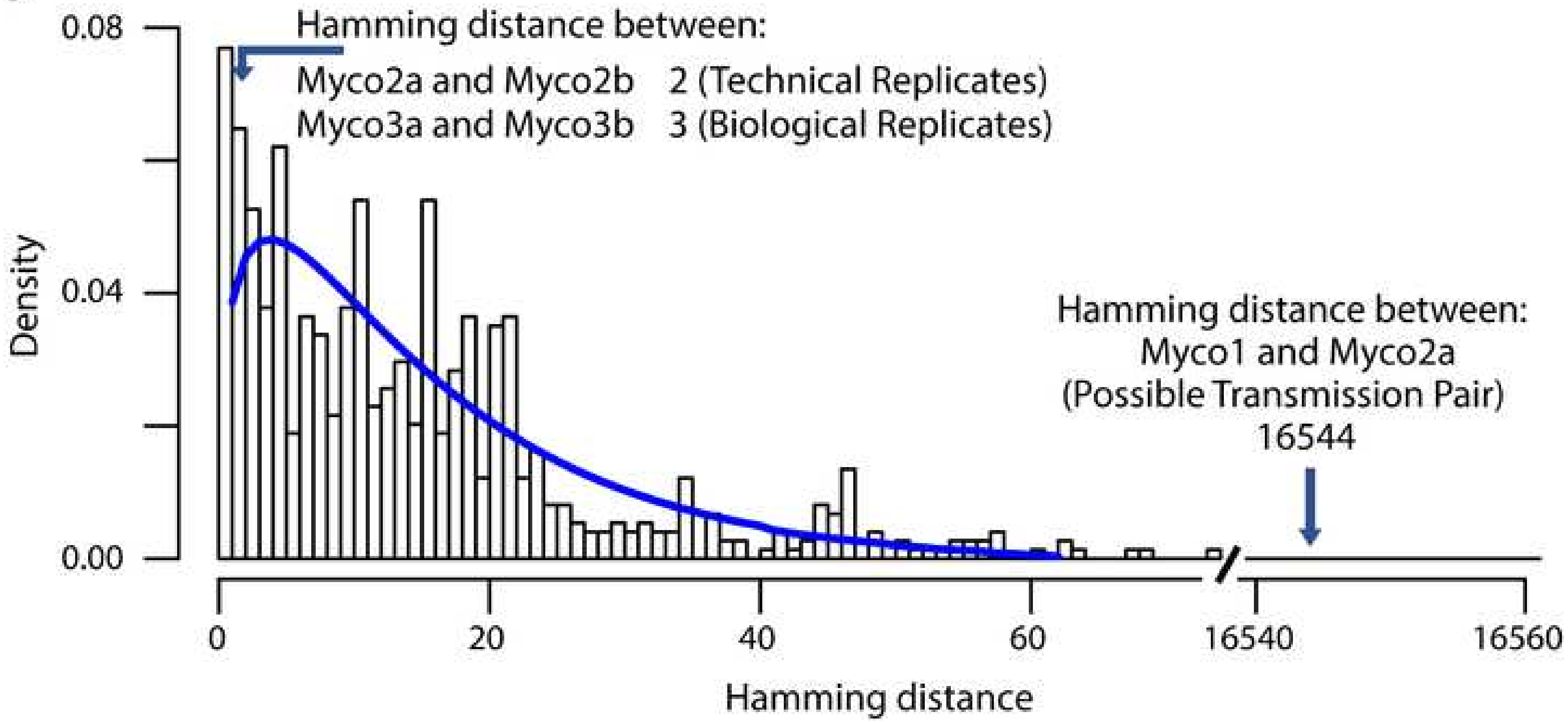

Click here to access/download;Figure;Fig3.tif

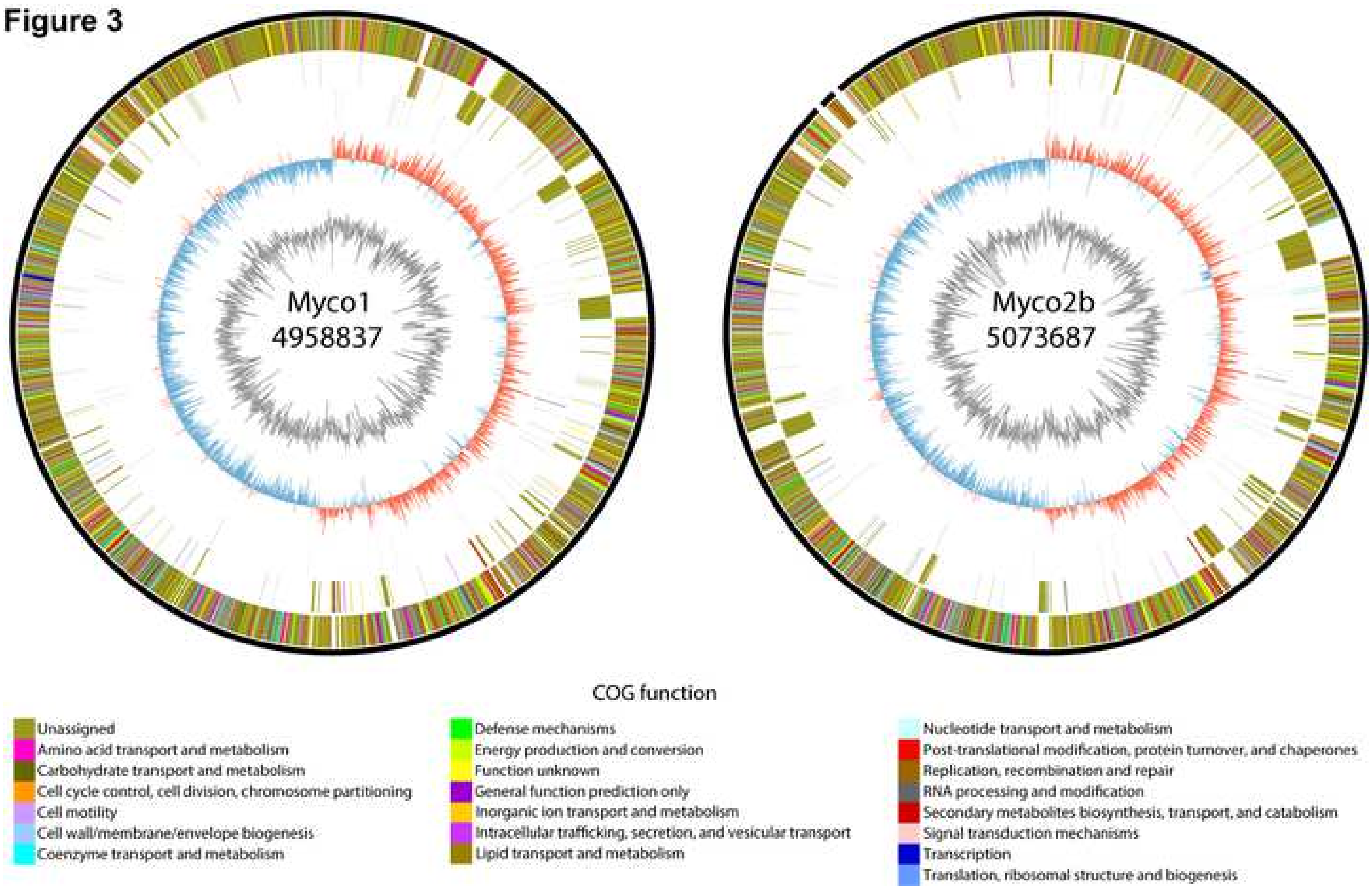

S1 Table

Click here to access/download
Supporting Information
S1Table_Genomes.xlsx

S2 Table

Click here to access/download
Supporting Information
S2Table_Vibrio.xlsx

S3 Table

Click here to access/download
Supporting Information
S3Table_Mycobacterium_resistance_genes.xlsx
