# Supplementary material for: Investigating hospital Mycobacterium chelonae infection using whole genome sequencing and hybrid assembly
Source: PLoS One. 2020 Nov 9;15(11):e0236533. doi: 10.1371/journal.pone.0236533 (PMC7652343; doi:10.1371/journal.pone.0236533)
Supplement: Attachment — Submitted filename: MycobacteriumRevResp.docx [file pone.0236533.s006.docx]

**Department of Microbiology**	Frederic Bushman, Ph.D.

3610 Hamilton Walk	Professor and Chair

426A Johnson Pavilion

Philadelphia, PA 19104-6076

Tel 215.573.8732 Fax 215.573.4856

[bushman@mail.med.upenn.edu](mailto:bushman@mail.med.upenn.edu)

www.med.upenn.edu

September 24, 2020

Revision of PONE-D-20-20588

Dear Madam or Sir,

Thank you very much for reviewing our paper “Investigating hospital *Mycobacterium chelonae* infection using whole genome sequencing and hybrid assembly”. We are gratified that the editors feel the manuscript has merit. Attached please find detailed responses to the reviewers’ comments, which we feel have greatly improved the paper. Following the editor’s recommendation, we have consolidated our responses to several of the reviewers’ points. In the below, the reviewer’s remarks are highlighted in yellow.

Reviewer 1.

We are very pleased that the reviewer wrote: “I believe you have some very interesting data here. The use of the V. campbellii isolate as a model to determine the viability of calling identity on fully sequenced and assembled isolates is really cool.”

First, we wish to thank the reviewer for the detailed comments on the manuscript. We have reworked the text extensively to take advantage of the reviewer’s efforts to improve the presentation. Changes are marked in the draft that includes tracked changes.

The reviewer wrote: “I think that some important points about your isolates and the comparison with the reference genomes are missing in the results as well as the discussion.”

To remedy this, we have marked the panels in Figure 1 so that the reference genomes used are fully identified in Table S1. We also mention a bit more about the genomes used for comparison in the discussion.

“A lot of the discussion comes off as a reiteration of the results.”

We have revised the Discussion extensively along the lines suggested by the reviewer.

Reviewer 2.

We are gratified that the reviewer wrote “The manuscript is very clear, the experiments have been conducted diligently with the appropriate controls and overall supportive conclusions.”

Minor
1) In Figure 1 a-b) it would be more useful to list all the species names or strain names.

We have numbered the strains used for comparison in Figure 1, and refer readers to Table S1 in the Figure 1 legend, where we provide accession numbers for all comparator strains.

2) In Figure 2) the resolution of the figure should be improved. As of now, it looks blurry image with the text font.

The blurriness comes from the PDF construction on the journal site. The figure drafts available by clicking the link on the upper right corner are higher quality. We have carefully checked each one to make sure this is the case.

3) In Figure 3) Circosplot, the genome size of Myco1 has shown as 4958837. But for the same, in Table S1) it is 4968398 bp update the right genome size. Represent the outer ring with the genome reference scale (0.5, 1, 1.5,… 5 Mb) that would help readers to identify a unique region precise position.

We have assured that the reported lengths match in the two places, and indicated a scale in the revised figure.

4) Table S1) Write all the species names, strain names, source names, and accession number of newly sequenced genomes (Myco1-7). Also, Update with the genome coverage information for the Oxford Nanopore and Illumina datasets.

We have updated Table S1 to include all of the above.

5) Ln 70, delete "that"

We have made the indicated edit.

6) Update software version numbers for all the tools. For example Prokka ver---, SamTools ver---, CheckM ver---

We have added the software version numbers as requested.

7) Ln 152, rewrite as " 100% conserved, likely due to the draft genomes".

Done

8) Ln 167, rewrite "45 to 64" instead "46 to 64"

Done

9) Table 2) Footnote: Also write complete abbreviations for R, S and I

Done

10) Ln 194, alignment to a reference genome (Myco1? it has not stated which was the reference was used to map SNVs as a reference).

We have specified the reference in the revision.

11) Ln 210, we used all 43 genomes (as of now at NCBI,47 genomes available) possibly mention the latest sequence retrieved date. For these 43 genomes, is it PROKKA annotation or the NCBI annotation that has been used?

We specify the date of the last genome retrieved, and that we used the Prokka annotation.

12) Ln 264, fewer core genes (3143 vs 3368). Not discussed how similar is this phylogeny compared to the hybrid assemblies phylogeny (if you have the data)? Fig 1a

We performed this comparison and found that the genomes were placed similarly. We included a sentence of this result.

13) Ln 270, "Providing strong evidence that they are different strains". possibly with the ANI values, this sentence makes strong evidence supporting different strains.

We have modified the indicated section to include the reviewer’s point.

14) Ln 356, a suggestion to delete "Our investigation of the genetic basis of M. chelonae drug resistance was inconclusive". Line 378-379, the statement provides candidate genes.

We have rephrased the indicated section as suggested.

15) It should be noteworthy, to discuss SNVs to their genomic positions such as is it belong to the coding region or intergenic sequence. Also, How many genes and which genes are affected, and their functional role.

We carried out this comparison and found nothing notable. We added a table of genes with the highest and lowest numbers of SNVs (new Table S2), but unfortunately we were unable to come up with any simple interpretation.

16) SNVs per MB site was discussed in this work. It should be appropriate to compare how this SNVs per MB is in other mycobacteria eg. NTMs or pathogenic mycobacteria.

We have added a paragraph to the Discussion comparing the frequencies SNVs per MB in various Mycobacterium strains.

We thank the editor and reviewers for their helpful comments, which we feel has greatly strengthened the manuscript. We hope the paper is now suitable for publication in PLoS ONE.

Sincerely,

Rick Bushman
